# Public acceptability of development in the Northern Forest of Vermont, USA—The influence of wildlife information, recreation involvement, and demographic characteristics

**DOI:** 10.1371/journal.pone.0203515

**Published:** 2018-12-17

**Authors:** Jessica L. Espenshade, James D. Murdoch, Therese M. Donovan, Robert E. Manning, Charles A. Bettigole, John Austin

**Affiliations:** 1 Rubenstein School of Environment and Natural Resources, University of Vermont, Burlington, Vermont, United States of America; 2 U.S. Geological Survey, Vermont Cooperative Fish and Wildlife Research Unit, University of Vermont, Burlington, Vermont, United States of America; 3 Ucross High Plains Stewardship Initiative, Yale School of Forestry and Environmental Studies, Yale University, New Haven, Connecticut, United States of America; 4 Vermont Department of Fish and Wildlife, Barre, Vermont, United States of America; University of Kwazulu-Natal, SOUTH AFRICA

## Abstract

Increasing development such as roads and houses will alter future landscapes and result in biological, social, and economic trade-offs. Managing development requires information on the public’s acceptability of development and understanding which factors shape acceptability. In this study, we examined three questions: 1) What is the public’s acceptability of development? 2) Is acceptability of development influenced by wildlife information? and 3) Is the maximum amount of acceptable development influenced by views about wildlife, involvement in outdoor recreation, and demographic factors? We conducted a visual-preference survey of 9,000 households in Vermont, USA that asked about acceptable levels of development, acceptability of wildlife, involvement in recreation, and individual and town demographics. The survey response rate was 44%. Maximum acceptable condition (MAC) for development was 41 houses/km^2^ and not meaningfully influenced by broader consequences of development on seven common wildlife species. MAC was influenced by views on individual species, including bear and coyote, but not by other species such as deer, fox, and bobcat. Respondents with a positive attitude toward bear favored less development, whereas the opposite relationship existed for coyote. Similarly, MAC was negatively influenced by involvement in birding and hunting, but not by other common recreational activities. Among demographic factors, respondents that were younger and not born in Vermont were more accepting of development. Population density also positively influenced development acceptability. Results provide measures of the public’s acceptability of development that can help guide decision-making about development, wildlife, and recreation management.

## Introduction

The world population is estimated to reach 9.7 billion people by 2050 [1] and higher human densities have led to increasing residential, commercial, and industrial development [2]. From 1982 to 2003, developed land in the United States increased by 48%, which equates to 680,000 ha of rural land converted annually [2, 3]. Development is expected to continue with an additional 10 million ha of forested land predicted to be converted by 2030 [2, 4]. Increasing development, especially of forested landscapes, can lead to the loss, degradation, and fragmentation of habitat and result in large cumulative effects for biodiversity, soil, air, and water quality [5, 6].

Changes in forest extent due to development are estimated to affect habitat quality in U.S. forests for up to 90% of all mammal, reptile, bird, and amphibian species in U.S. forests, many of which (28%) are endangered [7, 8]. For interior species and those sensitive to edge effects, forest fragmentation may have especially strong negative impacts on population size and patterns of distribution [7, 9, 10]. However, not all species are negatively affected by development. Development provides some species, such as raccoon (*Procyon lotor*) and red fox (*Vulpes vulpes*), with increased feeding opportunities, a variety of structures for shelter, and areas with fewer predators and competitors [5, 11, 12].

Development not only affects the abundance and distribution of wildlife, but also opportunities for recreation. Outdoor recreation, such as bird watching, hunting, and hiking, generally requires open undeveloped land for an acceptable recreation experience due to the value of scenic beauty [13, 14]. Satisfaction and enjoyment from a recreation experience may be directly correlated to the number of people seen or distance from development [15, 16]. For example, among hunters, the ability to connect with nature and escape from an urban environment are key components of the hunting experience, regardless of the actual hunting outcome [17, 18].

As development continues, impacts on wildlife and recreation can be mitigated on a large-scale by policy. Environmental policy, especially related to land-use planning and development, is largely shaped by public opinion [19]. Politicians often will not address environmental issues such as wildlife conservation without the support of public opinion and consequently, environmental policy is created around the passions, interests, and criticisms of the public [19]. Large policy changes are especially pervasive when there is a widespread public opinion shift on salient issues [20].

A greater understanding of public attitudes can help guide policy and landscape management strategies [19, 21]. If environmental issues are an important consideration in development planning, public perceptions and attitudes about natural resource management, specifically wildlife and outdoor recreation, can have a considerable impact on responses to management actions and support for future planning decisions [21]. Management will consequently become more effective when managers recognize the multifaceted relationship between public attitudes and environmental management [22]. Understanding the balance between personal preferences and environmental impacts will allow for appropriate and effective regulation that will meet the needs of diverse stakeholders over time [21, 22].

Information has been shown to influence public opinion when it is accessible, relevant, easily understood and credible [20]. As a result, information and fact sheets have been used to influence public opinion, especially on environmental issues such as water use, recycling, and park visitor conduct [23–25]. However, the presentation of information can have a large impact on human behavior. Simply listing factual information may not have the same effect on behavior change as a visual or story telling approach [25, 26]. Information needs to be framed in a way that connects to the individual on a personal and relevant basis [24],

There are several techniques for assessing public opinion [27]. Surveys are a common tool to assess opinions and have been used to examine land use changes such as alternative energy development [28]. Two widely used approaches to investigate preference are questionnaires and visual preference surveys. While both approaches capture public preferences, they also have short-comings. For example, using verbal questions only is often inadequate for describing landscape or environmental design, whereas visual surveys may lead to misinterpretation depending on scale and other visual aids [29]. Visual preference surveys, in particular however, can be used to estimate public acceptability of a given condition, such as acceptability of trash levels along a trail, acceptability of crowd levels at a national park [14], and acceptability of development within a town [30].

In 2011, Bettigole et al. [30] conducted a visual preference survey to examine public acceptability of development in Vermont, USA. The study [30] found that, on average, Vermonters were willing to accept an 11% increase in development in their towns. Here, we build on the results [30] by not only re-examining public acceptability of development in Vermont, which is part of the Northern Forest ecosystem (see below) and expected to experience increased development [31], but also examining how acceptability is affected by impacts on wildlife and participation in outdoor recreation, both of which are valuable interests to Vermont residents [32–34]We examined three questions: 1) What is the public’s acceptability of alternative levels of development? 2) Is acceptability of development influenced by wildlife presence information? and 3) Is the maximum amount of acceptable development influenced by views about wildlife, involvement in recreation, and demographic characteristics?

## Methods

### Study area

Vermont is the second least populated U.S. state (resident population = 626,088 in 2015) and covers approximately 25,000 km^2^ [35]. It is part of the Northern Forest ecosystem—a 30-million acre working landscape that is home to more than two million residents and stretches from eastern Maine through New Hampshire and Vermont and into northern New York. Over the past century, the population of Vermont has almost doubled in size [35]. Landscape development in Vermont mainly impacts forests, which represent the dominant land cover (79%) and are mostly owed by individuals, with just 20% of forests being public (i.e., municipal, state, and federal public lands) [36]. From 2003 to 2009 the amount of intact woodlands (> 50 acres) decreased by approximately 34,000 acres (or 4%), mostly due to the construction of dwellings [31]. Future development is projected to convert undeveloped land at a rate that is 2.6 times faster than the rate of population growth [37]. Development effects on wildlife populations and recreation are especially relevant in Vermont, where over 62% of the population is regularly involved in wildlife-related recreation and the state ranks third in the country for percent of public involvement in these types of activities [32].

### Survey

To address our research questions, we conducted a survey that involved sending a visual preference questionnaire (adapted from [30]) to 9,000 households in Vermont. The survey protocol underwent review by the Institutional Review Board (IRB) at the University of Vermont. We received an exemption from IRB.

**Fig 1:**
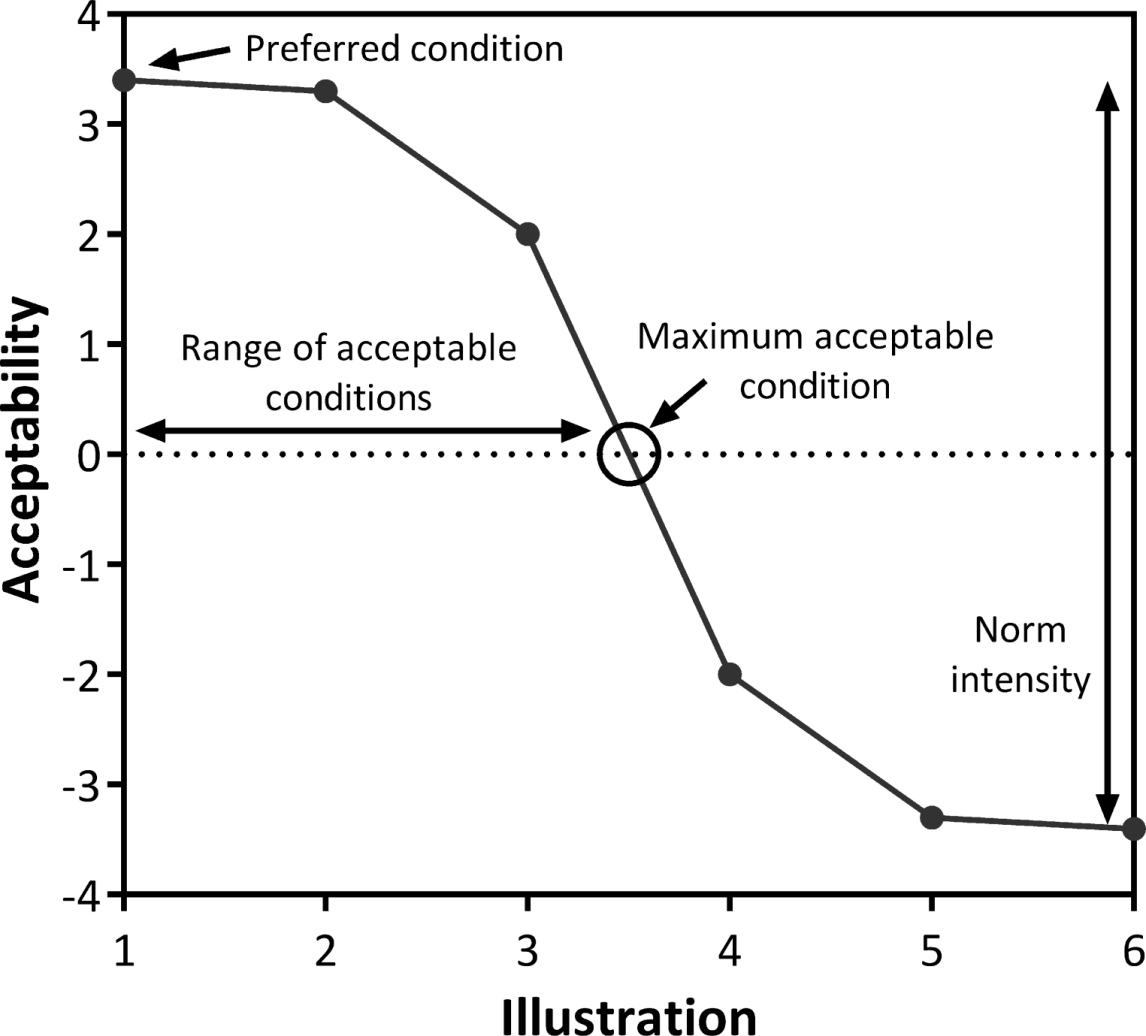
Example of a hypothetical social norm curve. Respondents to a visual preference survey scored their acceptability of a series of illustrations from -4 (completely unacceptable) to +4 (completely acceptable). Acceptability values represent average values among all respondents. The highest point on the curve is the preferred condition. Where the line crosses zero is the maximum acceptable condition (MAC) and the range of the values represents norm intensity.

The survey presented a series of six illustrations of a rural town visually altered to show increasing levels of development (Fig 1). Respondents were asked to rank each illustration on a scale of -4 to +4, with -4 indicating that the illustration was completely unacceptable and +4 indicating that the illustration was completely acceptable. Ratings allowed for the development of a social norm curve, in which the average acceptability value (y-axis) was plotted for each illustration (x-axis) (Fig 1;[15]). The range of acceptability scores within the curve indicated the intensity of interest in the measurement, and the point at which the curve crossed the neutral point of the x-axis was considered the maximum acceptable condition (MAC); it represented the point where respondent ratings fell out of the acceptable range and into the unacceptable range (Fig 1). Each of these curve characteristics allowed for a multidimensional understanding of public attitudes [14, 30, 38].

**Fig 2:**
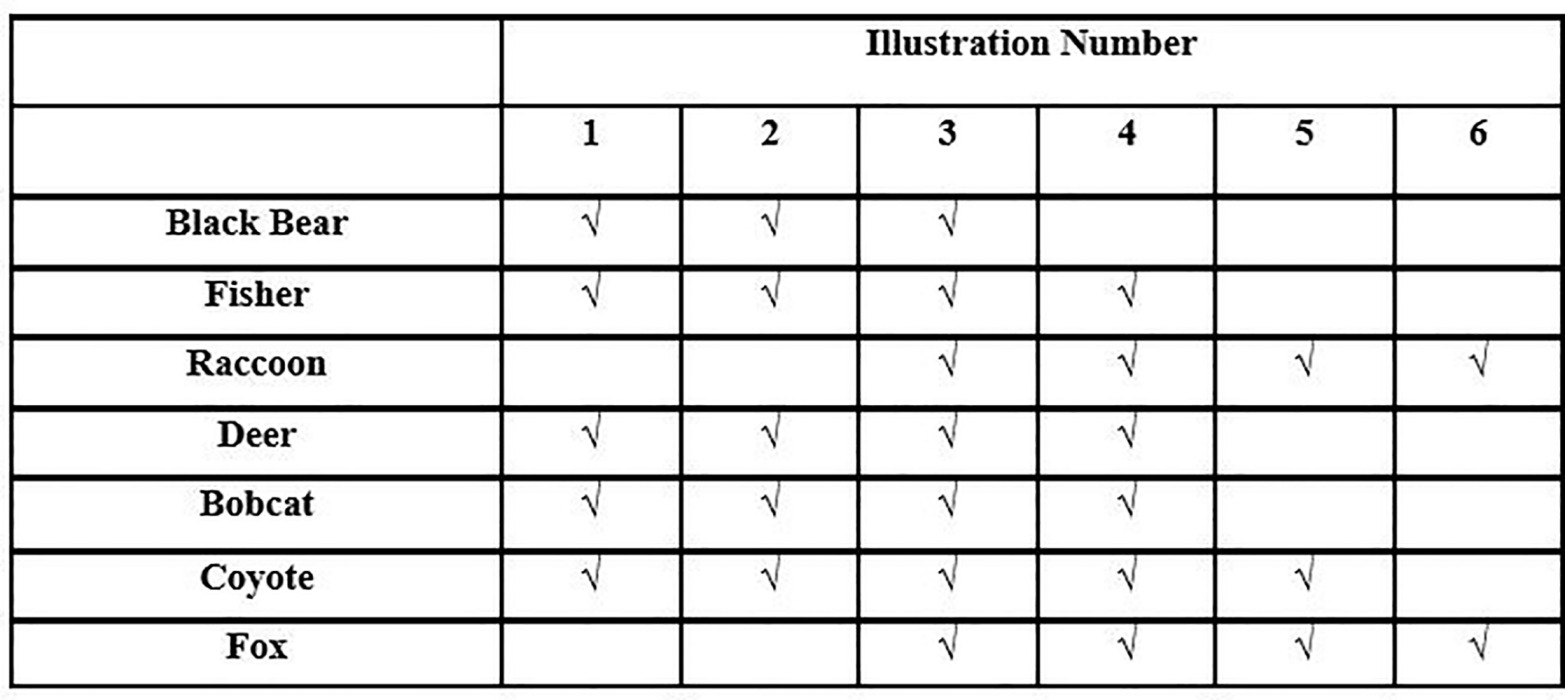
Wildlife information legend used in illustrations in the wildlife treatment group of a survey of development acceptability sent to households in Vermont, USA. A check mark indicated the species was present in the illustration. A blank space indicated that the species was not present in the illustration.

For Questions 1 and 2 (what is the public’s acceptability of development? and is acceptability of development influenced by wildlife presence information?), respondents were divided into two treatment groups: control and wildlife. The control group (*n* = 4,500) received the six illustrations from the Bettigole et al. [30] survey with no changes. Illustrations 1 to 6 in all surveys depicted the following housing densities: 1.71, 4.58, 12.57, 32.86, 88.06, and 235.83 houses/km^2^, respectively. The wildlife group (*n* = 4,500) received these same illustrations, but each illustration also included a legend with wildlife information (Fig 2). The legend included presence-absence information for each illustration on seven species, including black bear (*Ursus americanus*), bobcat (*Lynx rufus*), coyote (*Canis latrans*), deer (*Odocoileus virginianus)*, fisher (*Martes pennanti*), red fox (*Vulpes vulpes*), and raccoon (*Procyon lotor*). We selected these species because they are common and easily recognizable to the public [34]. We also only included one taxonomic group (mammals) to reduce variability. There was no description of the species or information about their ecological value included in the survey. As such we were unable to control for the effects of background knowledge about individual wildlife species, which could potentially influence responses [39].

The wildlife legend was developed from occupancy models derived from detection/non-detection data collected from 60 camera traps in the Champlain Valley of northwestern Vermont [40]. Occupancy modeling uses a multinomial maximum likelihood function to estimate model parameters while accounting for imperfect detection [41]. We developed a model set with 48 a priori models, applied the set to each species, and used Akaike’s Information Criterion (AIC) to rank each model [42]. Each top-ranking model was considered the best model in the set for each species. We then applied models to each pixel (30 x 30 m) of a National Land Cover Database (2006) map of each of the six illustrations using Geographic Information Systems (GIS) software (ArcGIS, v. 10, ESRI, Redlands, California, USA). We averaged occupancy probability across all pixels for each species, and considered the species ‘present’ at the mid-point after the ranges had been standardized across all species. The legend showed a check mark by the species if the species was determined to be present and a blank space if the species was absent (Fig 2). For details on the modeling approach, model rankings, and parameter estimates, see Espenshade [43].

To address Question 3 (is a respondent’s MAC score influenced by their views about wildlife, involvement in recreation, and demographic factors?), we asked all respondents in both the control and wildlife treatment questions related to wildlife, recreation involvement, and demographics and examined the influence of responses on MAC. For wildlife, individuals were asked, “How acceptable would it be to have the target species live in or near your town?” Respondents chose a number on the acceptability scale for each of the seven wildlife species. This question was asked to determine if there were differences in species acceptability, outside of the wildlife legend.

Recreation involvement was determined through the question, “Which of the following outdoor activities do you participate in?” Eight common outdoor recreation activities were listed, including birding, hiking, hunting, fishing, off-road ATVing, farming/gardening, snowmobiling, and camping. Respondents also had the opportunity to select “other” and write-in another activity or select “none of the above.” Demographic factors included questions about whether respondents were born in Vermont, whether they considered Vermont to be their primary residence, whether they were a home owner, and what year they were born.

### Survey sample design

We sent surveys to individuals between September and November 2014. Individuals were selected at random, but stratified by county size and treatment to have a representative statewide sample. Participants were required to be over 18 years of age, with no restrictions on demographic factors such as residence, ethnicity, homeownership, or gender. Mailing addresses were identified from ListGIANT (Westlake Village, California, USA). A pre-notification postcard was sent to all participants three days prior to the full survey. After the pre-notification postcard, the full survey was sent with identification numbers for tracking purposes. The full survey included an introductory letter, illustration packet, questionnaire sheet, and prepaid response envelope. Three days after the survey mailing a post survey reminder postcard was sent to the same individuals to encourage completion of the survey. Although response rates for mail surveys are often only 15 to 20% [44], token monetary payments have been shown to increase response by up to 50% [30, 44, 45].We therefore included a monetary incentive of one U.S. dollar to increase the response rate. Additionally, 1,800 of the non-respondents (20% of the original survey) received the entire survey again after a period of one month from the first survey mailing [44].

### Statistical analysis

To address Questions 1 and 2 (what is the public’s acceptability of development?, and is acceptability of development influenced by wildlife presence information?), we fit a social norm curve to each individual’s responses to the hypothetical development scenarios, in which we plotted the acceptability value among responses (y-axis) by illustration number (x-axis), which indicated how acceptability varied as development increased. To more precisely estimate the MAC, we fit a curve with a 3^rd^ degree polynomial to each individual’s responses. We then calculated the average MAC value among respondents to represent the public’s maximum acceptable level of development. We performed all statistical analysis using the R programming language [46].

For Question 3 (is the maximum amount of acceptable development influenced by views about wildlife, involvement in recreation, and demographic factors?), we used generalized linear mixed effect models to examine the influence of individual responses to wildlife, recreation, and demographic characteristics to their corresponding MAC value. We developed a separate model set for each category (wildlife, recreation, demographics) using an information-theoretic approach and model selection techniques (see below;[42]). For the wildlife models, we evaluated respondent’s MAC against their acceptability of each of the target wildlife species. For the recreation models, we evaluated respondent’s MAC against their participation (yes/no) in each of the eight recreational activities. For the demographic models, we evaluated the respondent’s MAC against five predictor variables, including the population density of the respondent’s county (Population), whether the respondent’s primary residence was in Vermont (VT Primary), whether the respondent was born in Vermont (VT Born), whether the respondent owned their home (Own House), and the year the respondent was born (Year Born). Using all subsets of additive combinations of variables, including the intercept model, we evaluated 113 models for wildlife, 497 for recreation, and 31 for demographic characteristics using the R package lme4 [47].

In all models, random effects were treatment group and respondent ID; all other covariates were considered fixed effects. We used model selection procedures to evaluate the relative support of models. We ranked models using Akaike’s Information Criterion (AIC) with bias-correction (AICc); these values were calculated using the R package MuMIn [48]. AICc can be used as a bias-correction for small sample sizes, but is used as the standard ranking in the MuMIn R package [48]. We considered models with *<* 2 ΔAICc to have strong empirical support [42].

## Results

The survey response rate was 44% (*n* = 3,629), after the non-deliverables (*n* = 724) were subtracted from the total number of questionnaires sent. The control treatment had a response rate of 45% (*n* = 1,846) and wildlife treatment had a response rate of 43% (*n* = 1,783). The second round of mailings had a response rate of 19% (*n* = 167) and 16% (*n* = 146) for control and wildlife, respectively. There were no statistical differences between first and second rounds of respondents for any of the research questions evaluated (p > 0.05). County response rates ranged from 40% to 46% for all 14 Vermont counties, which resulted in a representative sample geographically. A small number of respondents (1%) deleted their identification number and therefore could not be matched with location information, but were still included in the analysis (only county density information was missing from these respondents).

### Development

**Fig 3:**
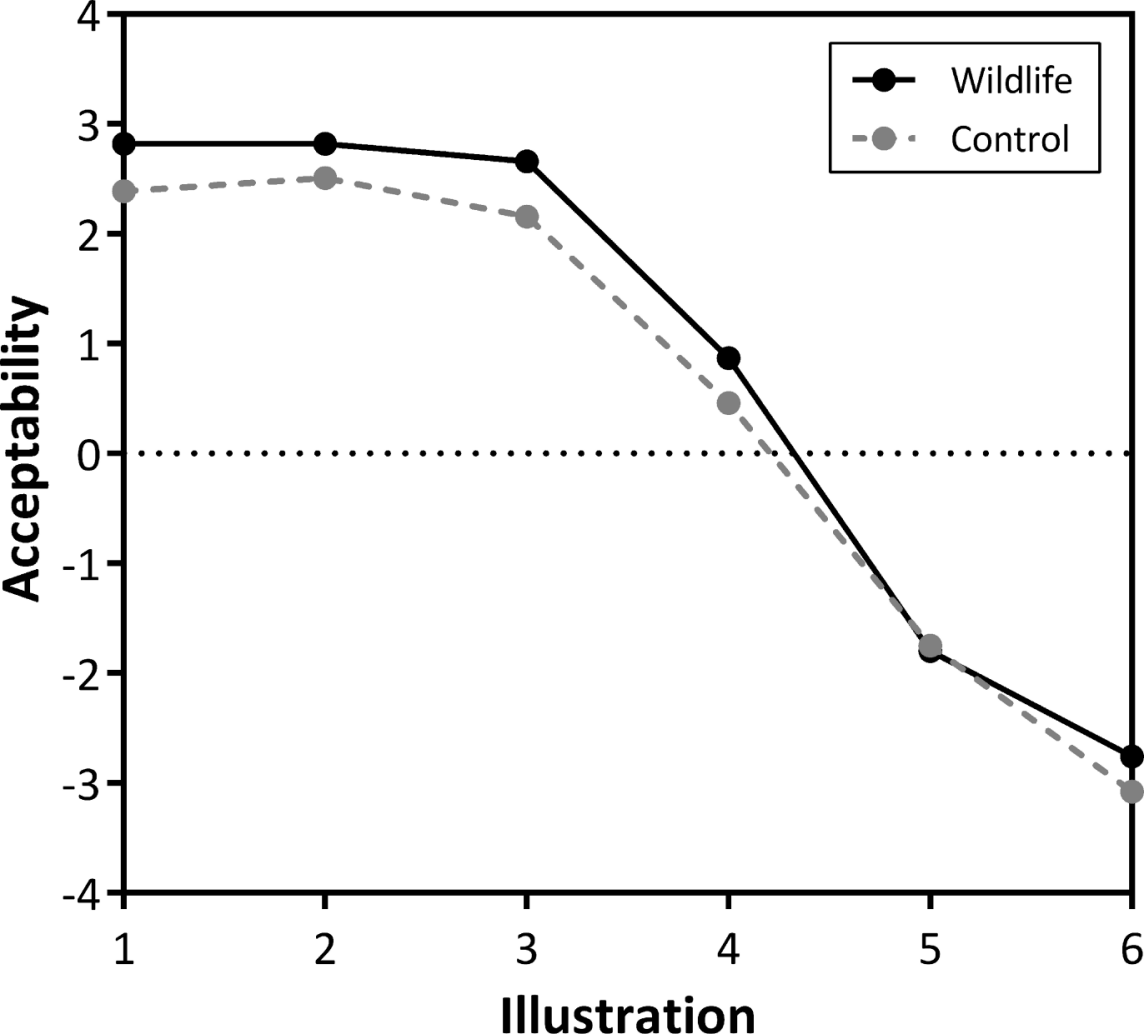
Social norm curves of acceptability of development by treatment group based on a visual preference survey sent to households in Vermont, USA. Respondents were asked to rate acceptability of six illustrations showing different amounts of development. Illustration 1 had the lowest amount of development (1.71 houses/km^2^) and subsequent illustrations increased exponentially in housing density to Illustration 6 (235.83 houses/km^2^). The ‘wildlife’ treatment represents responses to surveys (*n* = 1,783) that included information on the presence/absence of seven species in each illustration. The ‘control’ treatment represents responses (*n* = 1,846) to surveys that did not include wildlife information. Each value on the curves is the mean acceptability score across respondents. The maximum acceptability point, x when y is zero, was 4.19 (39 houses/km^2^) for control surveys and 4.30 (44 houses/km^2^) for wildlife surveys.

Survey data showed the highest level of acceptability for illustration 2 for both the control and wildlife treatments (Fig 3). The difference of means between the treatment groups was negligible: 0.42, 0.32, 0.50, 0.41, 0.06 and 0.30 for illustrations 1 through 6, respectively (Fig 3). Illustrations 1–4 were all considered acceptable for both treatments, with the mean MAC being 4.19 ± 0.02 SE (39 houses/km^2^) for control surveys and 4.30 ± 0.02 SE (44 houses/km^2^) for wildlife surveys. Illustrations 5 and 6 were considered unacceptable for both treatments. Mean MAC among all surveys (control and wildlife) was 4.22 ± 0.02 SE (41 houses/km^2^). Norm intensity was slightly higher for the wildlife group (5.58) compared to the control group (5.46).

### Wildlife

In response to the question, “How acceptable would it be to have the following animal live in or near your town?”, deer had the highest average level of acceptability (mean ± SE = 2.91 ± 0.03) among all wildlife species. Fox, raccoon, bobcat and bear had similarly positive levels of acceptability (mean ± SE = 1.80 ± 0.04, 1.51 ± 0.04, 1.20 ± 0.05, and 1.12 ± 0.05, respectively). Fisher and coyote had the lowest levels of average acceptability (mean ± SE = 0.52 ± 0.05 and 0.19 ± 0.05, respectively).

**Fig 4:**
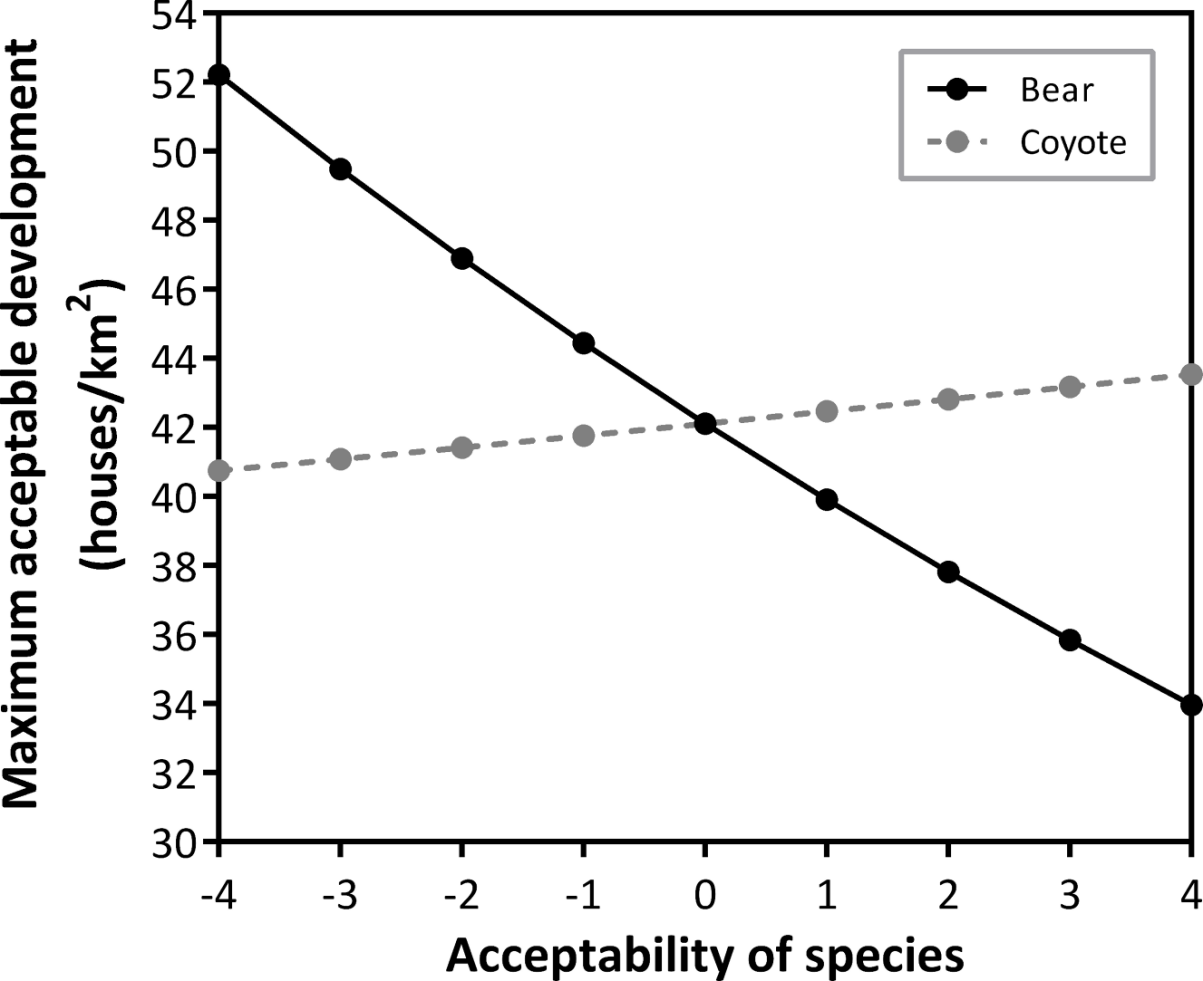
Effect of acceptability of bear and coyote on the maximum acceptable level of development in a respondent's town. Values based on a top-ranking model of responses to a visual preference survey sent to households in Vermont, USA. Species acceptability ranged from -4 (completely unacceptable) to +4 (completely acceptable).

Of the models that evaluated respondent’s MAC for development against their MAC values for each of the target species, only one had strong empirical support (Table 1). Covariates in this top model included acceptability of bear, fisher, raccoon and coyote (Table 1). Acceptability of bear had a negative influence on MAC for development, whereas acceptability of coyote had a positive influence on MAC for development (Fig 4). That is, as respondents’ acceptability of bear increased, the acceptability of development decreased, and as their acceptability of coyote increased, the acceptability of development increased. Confidence intervals (95%) for fisher and raccoon parameters overlapped zero indicating low precision in these estimates, so we did not consider them in our interpretation (Table 1).

**Table 1:** Relative ranking of models of the influence of wildlife views on acceptability of development based on responses to a visual preference survey of households in Vermont, USA. Respondents indicated wildlife views by rating the acceptability of each species living in and around their town on a scale from -4 (completely unacceptable) to +4 (completely acceptable). Wildlife views were used to predict acceptability of development on the same scale. Models were ranked based on Akaike’s Information Criterion (AICc) and those with ΔAICc of < 2 were considered to have strong empirical support. Only the top 5 model results are shown. Upper and lower 95% confidence intervals are listed in parentheses for parameter estimates in the top model.

Intercept	Bear	Bobcat	Coyote	Deer	Fisher	Fox	Raccoon	DF	AICc	ΔAICc
4.2480 (4.1614,4.3339)	-0.0546 (-0.0711,-0.0379)	-	0.0287 (0.0121,0.0456)	-	-0.0088 (-0.0258,0.0085)	-	0.0084 (-0.0099,0.0260)	7	8096.8	0
4.2580	-0.0528	-	0.0309	-	-0.0082	-	-	6	8101.1	4.27
4.2690	-0.0479	-0.0118	0.0319	-0.0061	-0.0065	-	0.0096	9	8108.0	11.18
4.2730	-0.0468	-0.0119	0.0344	-0.0030	-0.0057	-	-	8	8112.6	15.76
4.2280	-0.0478	-	-	-	0.0029	-	0.0162	6	8113.9	17.06

### Recreation

**Fig 5:**
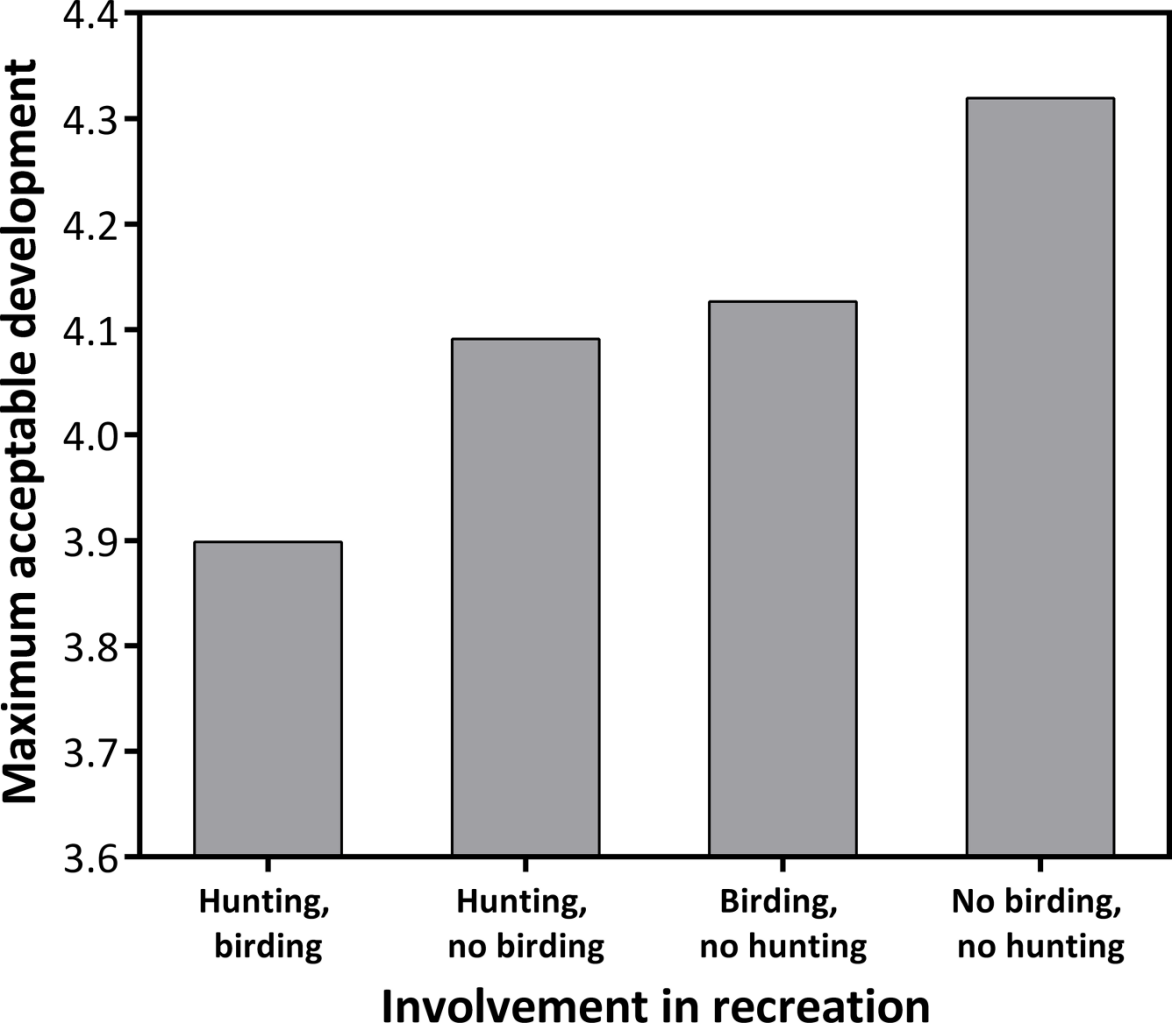
Effect of involvement in birding and hunting on the maximum acceptable level of development in a respondent's town. Values based on a top-ranking model of responses to a visual preference survey sent to households in Vermont, USA.

Nearly all respondents (95%) engaged in at least one of the recreation types indicated on the questionnaire. The majority of respondents considered themselves hikers (68%) or farmers/gardeners (67%). Snowmobiling (12%) and ATVing (13%) participants were the least numerous. Among the remaining recreation categories, 29% selected birding, 28% selected hunting, 39% selected fishing, and 42% selected camping. A small percentage (5%) selected ‘none of the above’ and 35% of respondents wrote-in additional recreational activities–most of which (16%) were skiing or biking. Only one recreation model had strong empirical support for predicting a respondent’s MAC score for development (Table 2). Covariates in this top model included participation in birding and hunting. Respondents who participated in these activities had a lower acceptability of development (Fig 5).

**Table 2:** Relative ranking of models of the influence of recreation participation on acceptability of development based on responses to a visual preference survey of households in Vermont, USA. Respondents indicated participation by checking a box next to each recreation type listed (*n* = 8). Models were ranked based on Akaike’s Information Criterion (AICc) and those with ΔAICc of < 2 were considered to have strong empirical support. Only the top 5 model results are shown. Upper and lower 95% confidence intervals are listed in parentheses for parameter estimates in the top model.

Intercept	ATVing	Birding	Camping	Farming	Fishing	Hiking	Hunting	Snowmobiling	None	DF	AICc	ΔAICc
4.3190 (4.234,4.405)	-	-0.1927 (-0.2619,-0.1572)	-	-	-	-	-0.2279 (-0.2985,-0.1572)	-	-	5	8184.7	0
4.3260	-	-0.1954	-	-	-	-	-0.2073	-0.1016	-	6	8187.0	2.31
4.2810	-	-0.2015	-	-	-	0.0573	-0.2263	-	-	6	8188.9	4.24
4.3190	-	-0.1928	-	-	-	-	-0.2280	-	-0.0019	6	8190.0	5.26
4.3210	-0.0325	-0.1935	-	-	-	-	-0.2183	-	-	6	8190.4	5.69

### Demographics

Slightly more than 99% of respondents considered Vermont to be their primary place of residence. However, only 47% of respondents were born in the state, with 91% of respondents owning their homes. The highest number of respondents (24%) came from Chittenden County (the most populous county; density = 113 people/km^2^). The lowest number of responses (1%) was from Essex County (the least populous county; density = 4 people/km^2^). The average year of birth among respondents was 1958, which equated to 57 years old. Respondent ages ranged from 18 to 92 years old.

Three of the models evaluating MAC for development against demographic factors had strong empirical support (Table 3). We considered the top-ranking model the best model in the set, but also report model average parameter values for each covariate (Table 3). The top model included the additive combination of county density, whether or not a respondent owned their home, whether or not the respondent was born in Vermont, and the year they were born (Table 3). Confidence intervals (95%) for the Own House covariate overlapped zero, and was thus not considered in our interpretation. County density had a positive effect on MAC: people who live in more populated areas had a higher acceptability of development. By comparison, being born in the state had a negative effect, indicating that native Vermonters were less accepting of development than others. Lastly, respondent age influenced development acceptability: those that were younger were more accepting of development than those that were older.

**Table 3:** Relative ranking of models of the influence of demographic characteristics on acceptability of development based on responses to a visual preference survey of households in Vermont, USA. Models were ranked based on Akaike’s Information Criterion (AICc) and only the top 5 model results are shown, along with model average parameter estimates. Upper and lower 95% confidence intervals are listed in parentheses for parameter estimates in the top models.

Intercept	Population	VT Primary	VT Born	Own House	Year Born	DF	AICc	ΔAICc
-18.0000 (-23.22,-12.74)	0.0050 (0.003,0.007)	-	-0.0940 (-0.157,-0.030)	0.0670 (-0.049,0.185)	0.0110 (0.009,0.014)	7	8020.2	0
-17.5600 (-22.79, -12.31)	0.0050 (0.003,0.007)	-	-	0.0720 (-0.044,0.190)	0.0110 (0.008,0.014)	6	8021.5	1.30
-17.7600 (-22.99, -12.49)	0.0050 (0.003,0.007)	-0.2400 (-0.569,0.087)	-0.0900 (-0.154,-0.026)	0.0720 (-0.044, 0.190)	0.0110 (0.009,0.014)	8	8021.9	1.67
-17.3100	0.0050	-0.2700	-	0.0780	0.0110	7	8022.5	2.35
-17.2800	0.0050	-0.2300	-0.0920	-	0.0110	7	8028.3	8.09
Model Average								
-17.6600	0.0050	-0.2600	-0.0920	0.0720	0.0110			

## Discussion

Increasing development, including construction of houses, roads, and infrastructure, will alter natural landscapes and have consequences for wildlife and nature-based recreation activities [5, 14, 16]. Our study examined three questions related to public acceptability of development in the state of Vermont and found that acceptability at 41 houses/km^2^ remained unchanged from a previous survey in 2011 [30] and that general information on the presence/absence of wildlife had little meaningful impact on acceptability levels. However, views on particular wildlife species influenced a person’s MAC for development in the Vermont landscape. Similarly, participation in some forms of recreation and demographic characteristics also affected an individual’s acceptability of development.

Even though development in Vermont slightly increased between 2011 and 2015 [35], the maximum acceptable level of development among Vermonters did not change [30]. The lack of change may reflect the inability of people to accurately interpret or respond to change in the environment or our ability to detect change through our survey approach. However, landscape development only occurred at a rate of 1% between 2011 and 2015, and it seems more plausible that this level of development was simply not meaningful enough to change people’s views.

Information on the consequences of an environmental change is often provided to the public as a means of influencing views and opinions [19, 20]. However, few studies have addressed the influence of information on the consequences of landscape change to wildlife. We found that the inclusion of basic information on the presence/absence of wildlife species under different scenarios of change did not have a meaningful impact on a person’s acceptability of development. There are several possible explanations for this result. First, wildlife may not be an important consideration for the general public, especially species that are generally considered to be widespread. This held true in our findings despite the fact that Vermonters are highly involved in wildlife-related activities relative to other states [32]. Second, the species we chose were all relatively common, and it is possible that people may have believed that these species would still persist in a developed landscape despite the information provided. Responses then favored the benefits of development over the potentially negative effects on common species. Lastly, the consequences to wildlife may have been an important consideration to respondents, but our survey approach did not adequately detect an effect of contextual information. The wildlife information presented in the figure legend may have been difficult to interpret or rationalize for some (e.g., one respondent commented that ‘red foxes were absent from one illustration that looks like my neighborhood, but I have seen the species many times before in my backyard’) and perhaps not detailed enough for others. Further studies should consider examining the effects of information type and presentation on responses. Our wildlife data were also presented without verbal reasoning or intentional behavior change messaging. Studies have indicated that data with narratives or messaging can influence conservation-oriented behavior [25, 26]. Our goal was not to influence behavior, but to objectively evaluate the effect of basic wildlife presence/absence information on views of development. The inclusion of additional messaging would have led to biases in our results, but may have value in future studies that aim to advocate for changes in landscape development.

Our survey and analysis did not account for the specific wildlife values held by respondents, which may have provided further insight into the responses to development scenarios. The value of wildlife can be broken into two broad categories including domination (view of wildlife that prioritizes human well-being over wildlife and treats wildlife in utilitarian terms) and mutualism (view of wildlife as capable of relationships of trust with humans and defined by a desire for companionship with wildlife) [21].Both place value on the existence and protection of wildlife, but could result in different levels of acceptability [49].For example, if a situation placed the value of wildlife above the needs of human society, domination value would be much lower than the mutualism value [21]. Consequently, development acceptability may depend on an individual’s personal views on the human-wildlife relationship.

Even though wildlife information presented in aggregate did not meaningfully affect an individual’s acceptability of development, individual views on some species including black bear and coyote did have an influence. The black bear is an iconic species of the Northern Forest ecosystem as an economically valuable game species and a symbol of wilderness important to both tourists and residents [50, 51]. Relative to coyotes, which are often considered a nuisance species [52], it is likely that respondents had favorable views on the value of black bears to Vermont. Raccoon and fisher also occurred in the top model, but confidence intervals around parameter estimates suggested that the effects of these factors were probably negligible. Interestingly, acceptability of deer was not a covariate in our top model. Deer are the most economically valued species in the state and arguably the most culturally important (the species adorns the state flag, even though it is not the official state animal; [18]. Efforts to influence public opinion on development may consider leveraging the favorability of black bear over other species. The relative importance of some species supports the notion that a relationship between individual value sets concerning wildlife and environmental concern exists [53, 54].

In our analysis of eight recreation activities, our model results indicated that only two activities, hunting and bird watching, influenced a respondent’s maximum acceptability of development. With the exception of fishing, these were the only wildlife-specific activities presented, so it is possible that respondents may have been more sensitive to the information presented on wildlife consequences in the survey illustrations. Numerous studies have shown that outdoor recreationists are more environmentally concerned in their value sets and exhibit greater pro-environment behaviors [16, 53, 55]. However, value differences often exist between those involved in consumptive recreation (e.g., hunting, fishing) and those involved in intrinsic, non-consumptive recreation (e.g., birding, hiking), which can influence views on landscape conservation [17]. Despite these differences, we found that birders and hunters essentially demonstrated the same opinions on development with both groups favoring less development. Our results confirm the findings of Cooper et al. [56] that examined whether wildlife recreationists are more likely to engage in pro-environment behaviors than non-recreationists in New York, USA. They found that both hunters and birdwatchers were 4–5 times more likely to engage in conservation behaviors than non‐recreationists and that hunter-birdwatchers (those that regularly participating in both activities) had the greatest likelihood of engaging in all types of conservation behaviors considered in the study (e.g., donating to local conservation efforts, enhancing wildlife habitat on public lands;[56]). Our results suggest individual views on development depend on involvement in birding and hunting in Vermont, which could inform policy at the intersection of wildlife management and landscape development.

All demographic covariates considered in our analysis had meaningful parameters estimates with the exception of home ownership, which had a negligible effect based on confidence intervals. Only the Vermont born factor (VT Born) was negative, indicating that in our study, native Vermonters were less accepting of development. Our results complement past research on developmental values. For example, respondents to interviews on wind farm development in Vermont believed that non-native residents brought a western urban centric mentality that did not recognize or appreciate aesthetic value over commercial value [57].Native Vermonters tend to view landscape development as a result of non-residents moving to the state and may view development as a challenge to their sense of place [58, 59].

The positive effect of county size on the MAC suggests that being surrounded by more development (e.g., in a highly populated county like Chittenden) may shift a person’s baseline of ‘normal’ development and lead to a higher level of acceptability [60]. Alternatively, those attracted to living in highly populated areas may simply have an inherent preference or lifestyle suited for a more developed landscape [61].

Lastly, MAC varied with respondent age as older individuals favored less development and younger individuals favored more development. This probably reflects changes in values about the quality of environment as one ages [58]. It may also reflect different generational baselines [60]. Younger respondents may be raised in a more developed landscape that older individuals (i.e., previous generations), and thus view those more developed conditions as ‘normal’ [60]. In addition, with the increased use of technology and disconnection with nature (especially among children), the loss and degradation of natural landscapes may be less noticeable and less alarming to future generations unless there is a change in value systems [62].

## Conclusions

As Vermont and other rural areas in the northeastern United States continue to be developed, it is important to consider that there is not just a space limit, but also a socially acceptability limit for development. In Vermont that limit is 41 houses/km^2^ based on the results of Bettigole el al. [30]and this study which is only 10% more than the current level of development in the state. Development planning has been primarily driven by economic factors. However, economics needs to be balanced with other factors that are important to residents of the state. For example, a public opinion survey of residents acknowledged the need for incorporating wildlife and landscape factors into decision-making aside from economics [33, 34].A majority of respondents (81%) in the survey indicated that wildlife habitat must be protected even if it reduces the land use options of some landowners and developers. Respondents also indicated that forest and habitat fragmentation (46%), fish and wildlife habitat loss (46%), and the loss of Vermont’s scenic landscape (41%) represented ‘big problems’[33, 34].

Our study provides greater insight into how wildlife and recreation shape views and opinions on development in the state. They suggest that aggregate information on wildlife presence/absence does not meaningfully influence development acceptability, but views on particular species, like black bear do. Similarly, involvement in two forms of recreation, one consumptive (hunting) and one non-consumptive (birding), influence levels of development acceptability. They also revealed the effects of certain demographic characteristics on acceptability.

Results from our study can help development planning at the town, country, and state levels. First, they provide a quantitative means (i.e., models) for estimating development acceptability based on demographic factors. Acceptability could be estimated given commonly available demographic information, which is useful for setting development goals, and tracked over time as demographics change. Models could also be used to predict acceptability under different scenarios of demographic change. Second, they indicate the importance of particular wildlife species and opportunities for recreation (in particular hunting and birding), which could inform where and how development occurs to ensure that these species and opportunities are not impaired. One approach to balancing ecological, economic, and social factors in the context of development is to adopt a multi-criteria decision making framework for landscape planning. A framework such as a simple multi-attribute ranking technique, assesses the consequences of various alternative actions (e.g., different areas to develop) on stated objectives (e.g., economic gain, wildlife occupancy, hunting and birding opportunities), which each have a goal (e.g., maximize or minimize) and weight that represent their relative priority [63]. Empirical data, expert opinion data, and input from the public can then be used to predict consequences and would allow managers to visualize how each alternative development scenario compares at achieving all objectives given their goals and weights. This approach has been successful in other contexts and could lead to more effective, balanced, data-driven decisions that meet the needs of the public and natural environment.
